# Angiogenic Serum Biomarker Levels Are Related to Onset of Labour in Low‐Risk Term and Post‐Term Pregnancies: A Prospective Observational Cohort Study

**DOI:** 10.1111/1471-0528.70231

**Published:** 2026-03-27

**Authors:** Ann‐Katrin Morr, Marc Baumann, Luigi Raio, Daniel Surbek, Anda‐Petronela Radan

**Affiliations:** ^1^ Department of Obstetrics and Gynecology, University Hospital Inselspital Bern University of Bern Bern Switzerland

**Keywords:** angiogenic biomarkers, labour onset, low‐risk pregnancies, post‐term, time‐to‐delivery

## Abstract

**Objective:**

To investigate whether angiogenic biomarkers at term in low‐risk pregnancies are associated with the timing of spontaneous and induced labour, and to assess changes from term to post‐term gestation.

**Design:**

Prospective, non‐interventional, observational cohort study.

**Setting:**

Single tertiary care centre, Switzerland.

**Population:**

Low‐risk term and post‐term pregnancies.

**Methods:**

Soluble fms‐like tyrosine kinase‐1 (sFlt‐1) and placental growth factor (PlGF) were measured and time intervals from sampling to delivery were analysed in spontaneous (*n* = 136) and induced labour (*n* = 48).

**Main Outcome Measures:**

Time to spontaneous labour onset and delivery; induction‐to‐delivery intervals; biomarker changes from term to post‐term.

**Results:**

In spontaneous labour, higher sFlt‐1 levels and sFlt‐1/PlGF ratios were inversely correlated with time to delivery (both *p* = 0.03). The sFlt‐1/PlGF ratio remained independently associated with shorter time to delivery after adjustment for gestational age, maternal body mass index, and parity. In induced labour, higher PlGF levels were associated with longer induction‐to‐delivery intervals (*p* = 0.02). From term to post‐term, PlGF declined (median 208 vs. 148 pg/mL, *p* < 0.0001), whereas sFlt‐1 (median 3128 vs. 3631 pg/mL) and the sFlt‐1/PlGF ratio (14 vs. 24) increased (both *p* < 0.0001).

**Conclusions:**

In low‐risk term pregnancies, an anti‐angiogenic profile is associated with shorter time to spontaneous delivery and increases from term to post‐term consistent with physiological placental maturation. In induced labour, biomarkers reflect placental state but do not independently predict induction dynamics. Their potential role in late‐term risk assessment warrants further investigation.

## 1 Introduction

Post‐term pregnancies are associated with increased adverse maternal and fetal risks, primarily due to placental ageing and declining function [1, 2, 3, 4]. Management requires balancing risks of expectant care against benefits of induction, guided by clinical surveillance including fetal heart rate monitoring, estimated fetal weight, and amniotic fluid volume. These indirect markers help assess short‐term fetal risk and guide delivery timing, but their predictive value is limited. Internationally, management of post‐term pregnancies is heterogeneous. Although international guidelines recommend offering induction of labour from 41 completed weeks in low‐risk pregnancies with reassuring findings, practice is increasingly individualised, balancing maternal and fetal risks and benefits and incorporating evidence such as the ARRIVE trial, which showed that elective induction at 39 weeks in low‐risk nulliparous women may reduce caesarean delivery without increasing adverse perinatal outcomes [5, 6, 7, 8, 9, 10, 11].

Circulating maternal biomarkers, particularly placental growth factor (PlGF) and soluble fms‐like tyrosine kinase‐1 (sFlt‐1), offer a promising biomarker‐based approach to assessing placental function. They may reflect placental stress and indicate its ‘biological age’ more accurately than gestational age [12, 13].

The sFlt‐1/PlGF ratio is an established clinical tool in managing preeclampsia and fetal growth restriction, both characterised by anti‐angiogenic imbalance [12]. High sFlt‐1/PlGF ratios and low PlGF concentrations are associated with preeclampsia, impaired fetal growth, and placenta‐mediated complications [14, 15, 16, 17, 18, 19, 20]. Emerging evidence also links these biomarkers to broader adverse outcomes, including intrapartum fetal compromise, spontaneous preterm labour, and unfavourable perinatal outcomes, even in low‐risk pregnancies [21, 22, 23, 24, 25, 26, 27].

Physiologically, PlGF rises during early pregnancy, peaks at 30 weeks, then gradually declines, while sFlt‐1 increases steadily with a steeper rise in the third trimester [28, 29, 30].

Despite robust evidence in preeclampsia and fetal growth restriction, the predictive value of angiogenic biomarkers in term and post‐term pregnancies remains unclear. Fiolna et al. found lower PlGF levels in adverse outcomes beyond 37 weeks, although neither PlGF nor sFlt‐1 alone predicted poor outcomes following induction [23]. A Norwegian study observed lower PlGF and higher sFlt‐1 in healthy post‐term pregnancies, provided gestational age‐specific reference ranges and noted oxidative placental stress with elevated anti‐angiogenic profiles. They also reported that pregnancies with reduced fetal movements and suspected placental dysfunction exhibited low PlGF, high sFlt‐1, and increased risk of adverse delivery outcomes [31, 32, 33, 34].

To date, no prospective study has examined correlations between angiogenic biomarkers at term and time to delivery in low‐risk pregnancies outside preeclampsia. Therefore, we assessed whether maternal sFlt‐1, PlGF and their ratio at term are associated with time to spontaneous or induced labour onset and delivery.

## 2 Material and Methods

This prospective, non‐interventional, single centre observational cohort study was conducted between September 2022 and September 2023 at the Department of Obstetrics and Fetomaternal Medicine, University Hospital of Bern, Switzerland.

All participants provided written informed consent.

Ethical approval was obtained from the Ethics Committee of the Canton of Bern, Switzerland on August 2nd 2022 (ethical approval number 2022‐00892).

Patients and the public were not involved in the study design, conduct, analysis or reporting.

The primary aim was to evaluate whether maternal angiogenic biomarkers (PlGF, sFlt‐1 and the sFlt‐1/PlGF ratio) at term in low‐risk pregnancies are associated with spontaneous labour onset, delivery timing, and induction‐related intervals.

Secondary outcomes included changes in biomarker levels from term to post‐term (two time‐points), associations with amniotic fluid volume (single deepest pocket) and the need for labour induction.

Outcomes were predefined based on study objectives and existing literature; no formal core outcome set was used.

Low‐risk pregnant individuals with normal fetal weight and amniotic fluid volume were enrolled at routine visits at term (defined as gestational age between 39 + 4 and 40 + 3 weeks).

The estimated date of delivery and thus gestational age was calculated according to Naegele's rule (estimated as 280 days from the first day of the last menstrual period) and confirmed by first‐trimester ultrasound. When ultrasound measurements differed by more than 5 days from the calculated gestational age, the estimated date of delivery was adjusted accordingly.

Exclusion criteria included preeclampsia/hypertensive disease, small for gestational age fetus/fetal growth restriction, abnormal amniotic fluid, fetal anomalies, severe maternal comorbidities, elective caesarean section, or age < 18 years.

Standard assessments included transabdominal ultrasound for fetal weight estimation and amniotic fluid volume measurement (single deepest pocket technique). Angiogenic biomarkers were assessed in all participants at the routine visit at term (between 39 + 4 and 40 + 3 gestational weeks), measuring maternal serum levels of PlGF and sFlt‐1 and calculating the sFlt‐1/PlGF ratio.

Participants subsequently requiring labour induction at a later time point had a second evaluation of angiogenic biomarkers at initiation of induction. To differentiate between term and post‐term sampling and to avoid overlapping gestational ages, the gestational age for the second biomarker assessment was defined as more than 40 weeks and 4 days.

Biomarker measurements were performed using Elecsys (electrochemiluminescence immunoassays, ROCHE Diagnostics, Rotkreuz, Switzerland). Investigators were blinded to biomarker results.

We analysed predefined time intervals from sampling to delivery separately for spontaneous and induced labour scenarios:

For spontaneous labour onset:

- total time from the first analysis to delivery (total time to delivery, TTDs),
- interval between analysis and labour onset (time to labour onset, TLOs)
- interval between labour onset and delivery (time from labour onset to delivery, TLDs).

Participants in the spontaneous labour group were stratified into those who presented with labour onset defined as regular contractions accompanied by rupture of membranes and/or cervical dilation ≥ 3 cm at hospital admission, and those who had irregular contractions consistent with the latent phase and subsequently progressed to labour after hospital admission.

For labour induction:

- total time from the first analysis to delivery (TTDi),
- interval between the first analysis and initiation of labour induction (time to labour induction, TLIi),
- interval between labour induction and labour onset (time from labour induction to labour onset, TLOi),
- duration of labour induction (time from labour induction to delivery, TLIDi).

Women in the induction group were admitted to hospital for initiation of labour induction prior to labour onset. Labour induction followed a standardised internal protocol, with mechanical methods, prostaglandins, and oxytocin applied singularly or in sequence based on cervical status and parity. Women with an unripe cervix (Bishop score < 6) underwent induction with either oral or vaginal prostaglandins or mechanical methods, followed by oxytocin once cervical maturation was achieved. Women with a favourable cervix (Bishop score ≥ 6) were induced directly with oxytocin [35].

We obtained clinical data concerning routine assessments, birth dates, maternal and neonatal outcomes from charts and electronic databases.

### 2.1 Statistical Analyses

The initial recruitment target reached 200 participants and was based on feasibility considerations and was chosen to allow exploratory assessment of small‐to‐moderate associations between angiogenic biomarkers and clinically relevant time‐to‐delivery intervals. After accounting for exclusions, the effective analysable sample size was 184 participants, providing approximately 77% power to detect an effect size of *f* = 0.20 in a regression model with one predictor and approximately 75% power to detect an effect size of *f* = 0.29 in more complex exploratory models at α = 0.05. Descriptive statistics summarised demographic and clinical characteristics (medians with interquartile ranges for continuous variables; counts and percentages for categorical variables). Comparisons between independent groups used the Mann–Whitney U test; paired data were analysed using the Wilcoxon signed‐rank test.

Associations between angiogenic biomarkers and time‐to‐delivery intervals were primarily assessed using Pearson correlation and simple linear regression, reflecting the exploratory aim of the study and to facilitate comparison with existing literature. Given the right‐skewed distribution of angiogenic biomarkers and time intervals, sensitivity analyses were additionally performed using Spearman rank correlation and linear regression models with natural log‐transformed outcomes. Analyses were performed separately for the spontaneous labour onset and induction group. Given the available sample size, we deliberately avoided highly parameterised models to reduce the risk of overfitting. Regression coefficients (β) are reported with 95% confidence intervals. All analyses were considered exploratory and hypothesis‐generating.

Diagnostic performance was additionally evaluated using receiver operating characteristic curve analysis (Area Under the Curve with 95% confidence intervals), with sensitivity, specificity, positive predictive value, and negative predictive value derived from 2 × 2 contingency tables.

All statistical analyses were conducted using GraphPad Prism version 10.4.0 for Windows (GraphPad Software, San Diego, CA, USA). A significance level of *p* < 0.05 was adopted.

## 3 Results

We included a total of 200 low‐risk pregnancies, which are hereafter described in two groups: 136 women with spontaneous labour onset and 64 women who required labour induction. The proportion of nulliparous women was 49% in the spontaneous labour group and 72% in the induction group. Table S1 summarises demographic and clinical characteristics, as well as birth and neonatal outcomes for both groups.

### 3.1 Spontaneous Labour Onset Group

Among 136 participants, PlGF and sFlt‐1 were measured once during routine prenatal care visit prior to labour onset at a gestational age between 39 + 4 and 40 + 3 weeks (Table S2).

**FIGURE 1:**
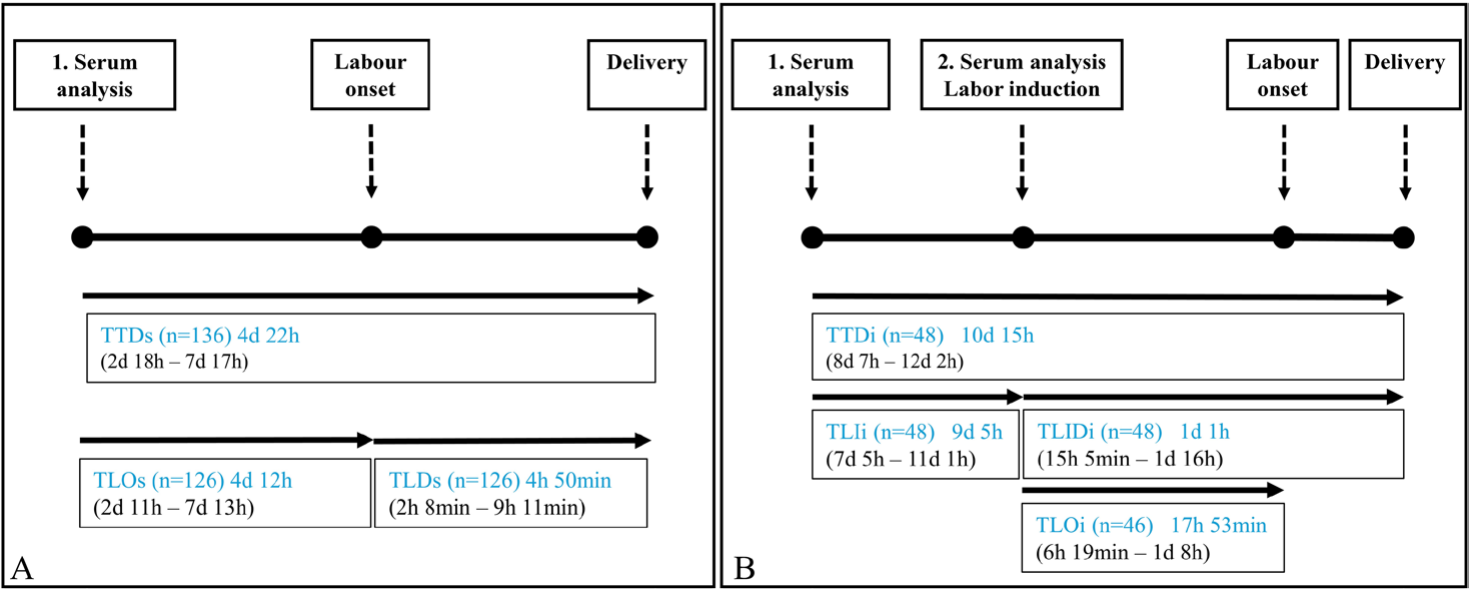
Time intervals for spontaneous labour onset group and labour induction group. (A) Spontaneous labour onset group. (B) Labour induction group. Continuous variables are presented as median (interquartile ranges). TLDs, time interval labour onset to delivery (spontaneous labour onset group); TLIDi, time interval labour induction to delivery (labour induction group); TLIi, time interval to labour induction (labour induction group); TLOi, time interval labour induction to labour onset (labour induction group); TLOs, time interval to labour onset (spontaneous labour onset group); TTDi, total time to delivery (labour induction group); TTDs, total time to delivery (spontaneous labour onset group).

As shown in Figure 1A, we recorded the median total time from biomarker sampling to delivery (TTDs, *n* = 136). In addition, we calculated the time intervals from sampling to labour onset (TLOs, *n* = 126) and from labour onset to delivery (TLDs, *n* = 126).

74/136 (54.41%) women were admitted with labour onset, presenting with regular contractions accompanied by rupture of membranes and/or cervical dilatation of at least 3 cm. 52/136 (38.23%) women presented with irregular contractions in the latent phase and subsequently progressed to labour after hospital admission. Additionally, 9/136 (6.61%) women were admitted for various reasons (non‐reassuring fetal heart rate monitoring, non‐cephalic presentation, suspected large‐for‐gestational‐age fetus, declined labour induction, or maternal request) and subsequently underwent pre‐labour caesarean delivery. In one case (0.74%), labour onset could not be determined due to delivery at an external hospital.

**FIGURE 2:**
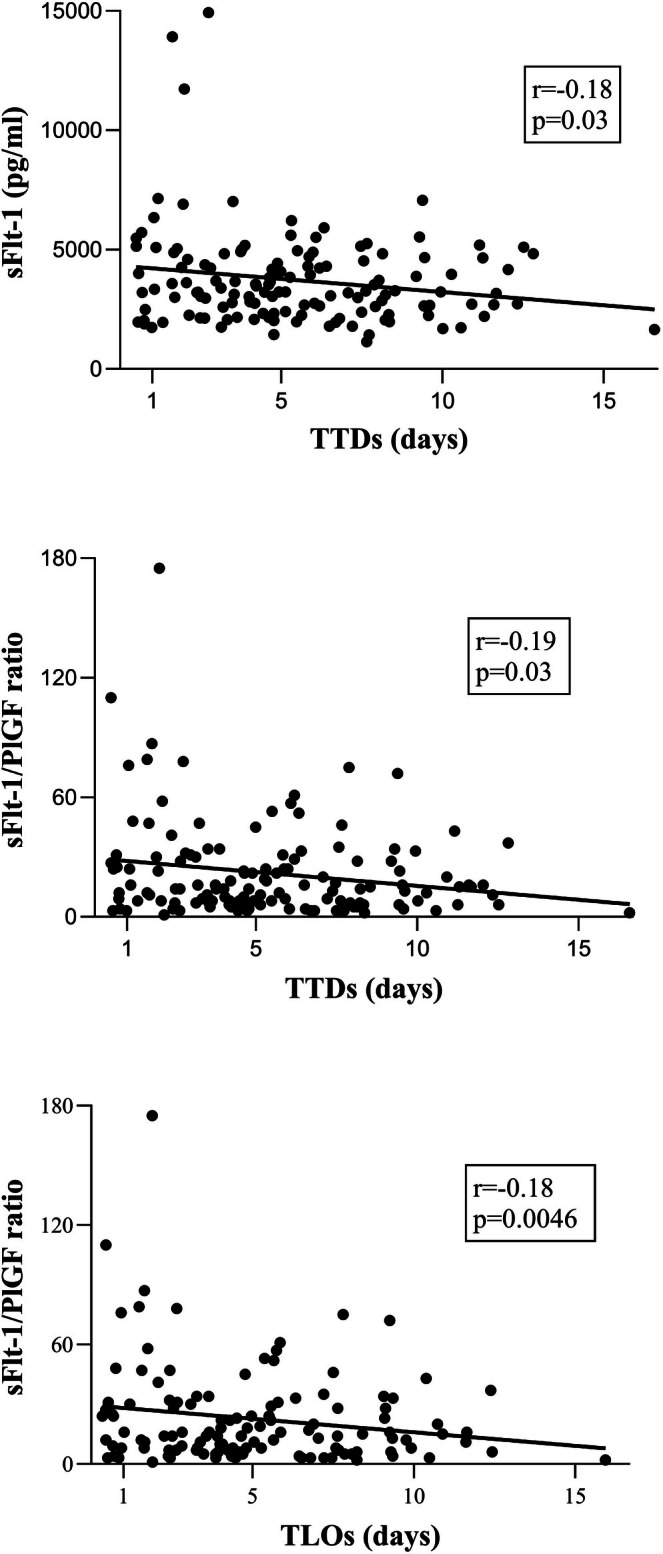
Pearson correlation analysis between total time to delivery/time to labour onset and sFlt‐1/PlGF ratio and sFlt‐1 in the spontaneous labour onset group. *p*, *p* value; PlGF, placental growth factor; *r*, Pearson correlation coefficient; sFlt‐1, soluble fms‐like tyrosine kinase‐1; TLOs, time to labour onset (spontaneous labour onset group); TTDs, total time to delivery (spontaneous labour onset group).

As shown in Figure 2 and Table S3, Pearson correlation analysis showed inverse association between total time to delivery and sFlt‐1 (*r* = −0.18, *p* = 0.03) and the sFlt‐1/PlGF ratio (*r* = −0.19, *p* = 0.03), indicating that higher sFlt‐1 or a higher sFlt‐1/PlGF ratio were associated with a shorter interval to delivery. In contrast, PlGF alone showed no correlation with total time to delivery. The sFlt‐1/PlGF ratio also correlated inversely with time to labour onset (*r* = −0.18, *p* = 0.046).

Stratified by gestational age, we observed inverse correlations between sFLt‐1/PlGF ratio and TTDs (*r* = −0.25, *p* = 0.02), respectively TLOs (*r* = −0.22, *p* = 0.04) ≤ 40 + 6 weeks (*n* = 94), but not ≥ 41 + 0 weeks (*n* = 42).

Spearman analyses were directionally consistent but not significant (sFlt‐1: ρ = −0.13, *p* = 0.12; sFlt‐1/PlGF ratio: ρ = −0.14, *p* = 0.10). Natural log‐transformed regression confirmed an inverse association between sFlt‐1/PlGF and total time to delivery (β = −0.0073, 95% CI −0.0128 to −0.0018; *p* = 0.0095). After adjustment for gestational age at blood sampling, body mass index and parity, the association remained significant (β = −0.0085, 95% CI −0.0120 to −0.0043; *p* = 0.0048).

Adjustment variables were not independently associated with delivery timing.

ROC analyses were performed to evaluate how well the sFlt‐1/PlGF ratio predicts time to delivery. Based on these analyses, a cut‐off of 55 for the sFlt‐1/PlGF ratio was selected because it provided high specificity for identifying women who are unlikely to deliver soon. A sFlt‐1/PlGF ratio below 55 correctly identified 95.4% of women who did not deliver within 7 days and showed similarly high performance for the absence of delivery within 2 and 4 days (Table S4). The corresponding ROC curves are not shown, as they do not provide additional information beyond the reported performance metrics.

### 3.2 Labour Induction Group

All participants requiring labour induction had two assessments of angiogenic biomarkers: the first at the routine visit at term (39 + 4 to 40 + 3 gestational weeks) and the second at initiation of induction (≥ 40 + 4 weeks). Both biomarker assessments were conducted prior to labour onset and not during active labour.

Of 64 women requiring induction, 48 met inclusion criteria for analysis. Sixteen were excluded: eight due to missing second biomarker sampling and eight because induction was performed at < 40 + 4 weeks, overlapping with the term sampling window. The remaining 48 women underwent labour induction primarily for post‐term pregnancy (*n* = 41) or for pre‐labour rupture of membranes without spontaneous contractions after 12–24 h (*n* = 7). Induction followed a standardised protocol using mechanical methods, prostaglandins, or oxytocin depending on cervical status. 21/48 (43.75%) women were induced with prostaglandins alone, 4/48 (8.33%) underwent mechanical induction, 7/48 (14.58%) received oxytocin alone due to a favourable cervix and 16/48 (33.33%) received a combination of either mechanical methods or prostaglandins followed by oxytocin. Two women did not enter labour and were delivered by caesarean section.

Figure 1B (labour induction group) shows the median time intervals between the two biomarker samplings and beginning of labour induction, labour onset, and delivery. The median time between the first sampling at term and the second sampling performed at the beginning of labour induction (TLIi) was 9 days and 5 h.

When comparing the spontaneous labour onset group (*n* = 136) with the labour induction group (*n* = 64), no significant differences were observed in maternal serum PlGF, sFlt‐1 or the sFlt‐1/PlGF ratio at the first sampling at term (Table S2).

Within the induction group (*n* = 48), PlGF declined and sFlt‐1 and the sFlt‐1/PlGF ratio increased between first sampling at term and second sampling at the start of induction post‐term (Table 1). These changes align with a concurrent reduction in amniotic fluid volume.

**TABLE 1:** Comparison of angiogenic biomarkers and amniotic fluid at sampling 1 and 2 in the labour induction group.

	1. Sampling at term 40.0 gw (*n* = 48)	2. Sampling at labour induction 41.1 gw (*n* = 48)	*p*
PlGF, pg/mL	207.5 (133–5361)	148 (109–291)	< 0.0001
sFlt‐1, pg/mL	3128 (2782–4398)	3631 (2911–5051)	< 0.0001
sFlt‐1/PlGF ratio	14 (9–30)	24 (10–44)	< 0.0001
Single deepest amniotic fluid pocket, cm	5.0 (3.9–6.2)	4.1 (2.9–5.5)	0.007

*Note:* Continuous variables are presented as median (interquartile ranges).

Abbreviations: gw, gestational weeks; PlGF, placental growth factor; sFlt‐1, soluble fms‐like tyrosine kinase‐1.

**FIGURE 3:**
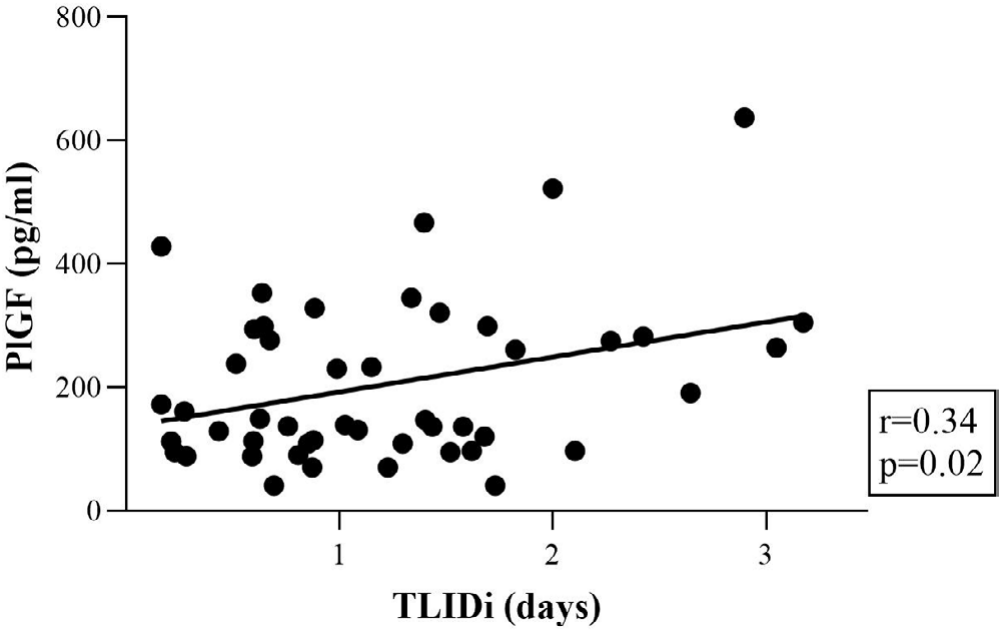
Pearson correlation analysis between time from labour induction to delivery and PlGF in the labour induction group. *p*, *p* value; PlGF, placental growth factor; *r*, Pearson correlation coefficient; TLIDi, time interval labour induction to delivery (labour induction group).

Using Pearson correlation analysis, higher PlGF was associated with longer induction‐to‐delivery time (TLIDi) (*r* = 0.34, *p* = 0.02). No other significant correlations were observed (Figure 3 and Table S3).

In multivariate analysis adjusting for cervical ripeness (Bishop score < 6 vs. ≥ 6) and gestational age at induction, PlGF was not associated with time to labour onset (β = 0.00062, 95% CI −0.00124 to 0.00249; *p* = 0.50). Cervical ripeness showed a strong inverse association with time to labour onset (β = −1.80, 95% CI −2.42 to −1.18; *p* < 0.0001), indicating significantly shorter time to labour in women with a ripe cervix. Gestational age at induction was not significantly associated with time to labour onset (β = 0.021, 95% CI −0.071 to 0.113; *p* = 0.65). The model explained approximately 49% of variance (*R* ^2^ = 0.49).

## 4 Discussion

### 4.1 Main Findings

Our study has four principal findings. First, angiogenic biomarkers, specifically sFlt‐1 levels and the sFlt‐1/PlGF ratio at term, were inversely correlated with total time to delivery in low‐risk pregnancies with spontaneous labour onset, indicating that elevated anti‐angiogenic markers are associated with shorter delivery intervals. Second, we observed significant changes in angiogenic biomarkers from term to post‐term, with PlGF declining and sFlt‐1 and the sFlt‐1/PlGF ratio rising. Third, during labour induction, higher PlGF at induction was associated with longer induction‐to‐delivery intervals. Finally, at term, no significant differences in sFlt‐1, PlGF or the sFlt‐1/PlGF ratio were observed between spontaneous and induced labour.

### 4.2 Strengths and Limitations

Strengths include the prospective design and focus on a well‐defined low‐risk cohort, minimising confounding and the use of standardised, validated assays for reliable biomarker measurements.

Limitations include a relatively small sample size, especially for labour induction. Biomarkers were assessed at predefined time points rather than longitudinally, so observed changes reflect between‐timepoint and gestational‐age differences rather than continuous trajectories.

Although correlations between sFlt‐1, the sFlt‐1/PlGF ratio and labour timing were statistically significant, they were moderate in strength, limiting predictive utility. Generalisability is limited by the highly selected, low‐risk population in a high‐resource healthcare system with lower baseline risks for adverse outcomes than other global obstetric populations. Consequently, extrapolation to broader or higher‐risk settings should be cautious and further studies in more heterogeneous cohorts are needed to determine clinical relevance.

### 4.3 Interpretation

To our knowledge, this is the first study to assess maternal sFlt‐1 and PlGF at term in relation to labour timing in low‐risk pregnancies outside preeclampsia, including spontaneous labour onset and delivery, induction‐to‐delivery intervals, and the need for labour induction, as well as biomarkers course in post‐term pregnancies.

We found that higher anti‐angiogenic biomarkers correlate with shorter delivery intervals, supporting a physiological anti‐angiogenic shift preceding labour [12, 13, 36]. This aligns with previous reports: Farina et al. showed higher sFlt‐1/PlGF ratios at 36 weeks predicted earlier spontaneous labour in low‐risk pregnancies [27]. Palma Dos Reis et al. linked the sFlt‐1/PlGF ratio to timing and mode of delivery in suspected preeclampsia [24]. Musilova et al. found elevated sFlt‐1 and sFlt‐1/PlGF ratio and lower PlGF in women delivering within 7 days of spontaneous preterm birth (23 and 35 weeks) [37]. In our multivariate analysis, higher sFlt‐1/PlGF ratios at spontaneous labour remained associated with shorter time to delivery, after adjusting for gestational age, maternal body mass index and parity, suggesting angiogenic imbalance may reflect aspects of biological readiness for parturition beyond chronological gestation.

Our study extends these findings to post‐term pregnancies, indicating that angiogenic biomarkers may detect subtle placental changes even in uncomplicated cases. Comparisons are limited by differences in study design, population, and gestational age: Farina et al. and Musilova et al. focused on earlier gestational ages, while Palma Dos Reis et al. studied a high‐risk population with suspected preeclampsia and typically altered angiogenic profiles.

Dynamic changes observed—PlGF decline and sFlt‐1/PlGF ratio rise—support a progressive anti‐angiogenic shift with advancing gestation in uncomplicated pregnancies [12, 13]. These observations are consistent with the work of Mitlid‐Mork et al., who reported elevated sFlt‐1 and reduced PlGF in post‐term pregnancies, together with histological evidence of placental ageing and vascular dysfunction despite the absence of overt pathology [31, 33]. Bowe et al. similarly linked altered angiogenic profiles in post‐date pregnancies to adverse delivery outcomes, likely of placental origin [32, 34]. Taken together, these findings suggest that even normal post‐term pregnancies may be accompanied by gradual subclinical placental ageing, characterised by an anti‐angiogenic shift. Thus, late‐third‐trimester angiogenic profiles may reflect not only pathological conditions such as preeclampsia, but also physiological placental senescence that could influence labour and birth outcomes. In this context, the term ‘low‐risk’ in our cohort refers to the absence of predefined obstetric or medical complications at inclusion and should not be interpreted as the absence of residual perinatal risk. Accordingly, the observed anti‐angiogenic shift with advancing gestation does not imply inevitable placental failure. Rather, it may reflect gradual placental maturation and a progressive decline in functional reserve that, in most low‐risk pregnancies, remains clinically compensated. In labour induction, higher PlGF at induction was associated with longer induction‐to‐delivery intervals univariably, suggesting that a more pro‐angiogenic profile may reflect a biological state that is less primed for transition to labour. After adjustment for cervical ripeness and gestational age, PlGF was not associated with time to labour onset after induction. In contrast, cervical ripeness showed a strong and independent association with time to labour onset, underscoring the dominant role of local cervical conditions in determining induction efficiency.

Angiogenic biomarkers and cervical status thus capture complementary aspects of parturition: PlGF may reflect placental maturation and systemic labour readiness, while cervical ripeness determines responsiveness once labour is initiated. Angiogenic biomarkers in induction should be interpreted as indicators of placental state, not triggers of delivery.

A secondary aim was to evaluate whether angiogenic biomarkers at term predict the need for labour induction. We observed no differences between women with spontaneous labour and those requiring induction, suggesting that these biomarkers alone are insufficient to distinguish labour onset in low‐risk pregnancies. This likely reflects the heterogeneous indications for induction and is consistent with Bowe et al., who reported limited predictive value of post‐date angiogenic imbalances for labour outcomes unless combined with additional clinical or placental parameters [33]. Most studies of sFlt‐1 and PlGF have focused on preeclampsia and other placental disorders with fewer examining their potential involvement in physiological term labour. Our findings suggest that angiogenic biomarkers may reflect normal placental maturation and late gestational adaptation in low‐risk term and post‐term pregnancies. Rather than serving as standalone predictors, they may contribute to a broader framework linking placental ageing with timing of parturition.

Although trials such as ARRIVE support elective induction at term, expectant management beyond the date of delivery remains common and is often influenced by patient preference [11]. Angiogenic biomarkers measured in late‐term pregnancy could potentially contribute to individualised counselling regarding expectant management versus induction. In women beyond 40 weeks with reassuring clinical findings but concern about placental reserve, a pronounced anti‐angiogenic profile may justify closer surveillance or earlier delivery, whereas a more balanced profile may provide additional reassurance during short‐term expectant management. Such an approach would complement, not replace, established clinical assessment and should be validated prospectively in larger, diverse cohorts before clinical implementation.

## 5 Conclusion

In this low‐risk term cohort, maternal sFlt‐1 and the sFlt‐1/PlGF ratio were inversely correlated with time to delivery, supporting the concept that an anti‐angiogenic shift accompanies the approach of parturition. From term to post‐term, rising sFlt‐1 and declining PlGF levels may reflect placental ageing and a progressive anti‐angiogenic state. In labour induction, higher PlGF levels were associated with longer induction duration, suggesting that a more angiogenic environment may correspond to slower labour progression. Biomarker levels at term did not differ between spontaneous and induced labour, limiting their utility as standalone predictors of induction need. Overall, angiogenic biomarkers appear to reflect late‐gestational placental maturation and systemic readiness for labour. Their clinical relevance in low‐risk term and post‐term pregnancies warrants further investigation, particularly in combination with established clinical parameters.

## Author Contributions

**Ann‐Katrin Morr:** conceptualisation, methodology, investigation; data acquisition; project administration; formal analysis; visualisation; writing – original draft preparation, writing – review and editing. **Marc Baumann:** conceptualisation; formal analysis; supervision; writing – review and editing. **Luigi Raio:** supervision; writing – review and editing. **Daniel Surbek:** conceptualisation; methodology; formal analysis; supervision; writing – review and editing. **Anda‐Petronela Radan:** methodology; investigation; data acquisition; formal analysis; supervision; writing – review and editing. All authors approved the final version of the manuscript.

## Funding

Internal Research grant, Department of Obstetrics and Fetomaternal Medicine, University Hospital Inselspital Bern, Switzerland.

## Ethics Statement

The study was approved by the Ethics Committee of the Canton of Bern, Switzerland on August 2nd 2022 (ethical approval number 2022‐00892).

## Consent

All participants provided written informed consent.

## Conflicts of Interest

The authors declare no conflicts of interest.

## Supporting information


**Table S1:** Demographic, clinical characteristics, birth data and neonatal outcome.

**Table S2:** Angiogenic biomarkers and amniotic fluid at sampling at term.

**Table S3:** Pearson correlation analysis between individual time intervals and angiogenic biomarkers for spontaneous labour onset group and labour induction group.

**Table S4:** Contingency table for cut‐off: sFlt1‐PlGF ratio 55.

## Data Availability

The dataset generated and analysed during this study is available from the corresponding author on reasonable request.
