# Supplementary material for: Angiogenic Serum Biomarker Levels Are Related to Onset of Labour in Low‐Risk Term and Post‐Term Pregnancies: A Prospective Observational Cohort Study
Source: BJOG. 2026 Mar 27;133(9):1777–84. doi: 10.1111/1471-0528.70231 (PMC13419333; doi:10.1111/1471-0528.70231)
Supplement: Supplementary file 1 — Table S1: Demographic, clinical characteristics, birth data and neonatal outcome. [file BJO-133-1777-s002.docx]

Table S1: Demographic, clinical characteristics, birth data and neonatal outcome

|  | **Spontaneous labour onset**  **(n = 136)** | **Labour induction**  **(n = 64)** |
| --- | --- | --- |
| Primiparous woman | 66 (48.5%) | 46 (71.8%) |
| Multiparous woman | 70 (51.5%) | 18 (28.2%) |
| Maternal age, years | 33 (30-35) | 34 (30.3-36) |
| BMI, kg/m^2^ | 27.3 (25.2-30.4) | 30.0 (26.5-32.8) |
| Ethnicity  White  Black  South Asian  East Asian  Mixed | 130 (95.7%)  4 (2.9%)  0  1 (0.7%)  1 (0.7%) | 59 (92.1%)  2 (3.1%)  1 (1.6%)  1 (1.6%)  1 (1.6%) |
| Smoking | 3 (2.2%) | 4 (6.3%) |
| Conception  Spontaneous  Hormonal stimulation/insemination  In vitro fertilization | 130 (95.6%)  3 (2.2%)  3 (2.2%) | 60 (93.7%)  0  4 (6.3%) |
|  |  |  |
| Gestational age at first sampling, weeks | 40.0 (39.9-40.0) | 40.0 (39.9-40.0) |
| Gestational age at second sampling, weeks |  | 41.4 (41.0-41.5) |
| Gestational age at delivery, weeks | 40.7 (40.4-41.0) | 41.6 (41.1-41.7) |
| Indication for labor induction  Post-term pregnancy  Rupture of membranes without active labour onset |  | 41 (85.4%)  7 (14.6%) |
| Mode of delivery  Vaginal delivery  Vaginal operative delivery  Secondary caesarean section  Primary caesarean section | 91 (66.9%)  19 (14.0%)  22 (16.2%)  4 (2.9%) | 20 (41.7%)  8 (16.6%)  20 (41.7%)  0 |
| Birthweight, g | 3608 (3336-3884) | 3663 (3350-3899) |
| Placental weight, g | 580 (513-650) | 582 (492-674) |
| 5 minutes Apgar score < 7 | 1 (0.7%) | 1 (2.1%) |
| Arterial cord pH < 7.10 | 6 (4.4%) | 3 (6.3%) |
| Neonatal intensive care unit admission | 3 (2.2%) | 1 (2.1%) |

Continuous variables are presented as median (interquartile ranges) and categorical variables as frequencies (percentages).

Abbreviations: BMI, body-mass-index
