# Supplementary material for: Angiogenic Serum Biomarker Levels Are Related to Onset of Labour in Low‐Risk Term and Post‐Term Pregnancies: A Prospective Observational Cohort Study
Source: BJOG. 2026 Mar 27;133(9):1777–84. doi: 10.1111/1471-0528.70231 (PMC13419333; doi:10.1111/1471-0528.70231)
Supplement: Supplementary file 2 — Table S2: Angiogenic biomarkers and amniotic fluid at sampling at term. [file BJO-133-1777-s001.docx]

Table S2: Angiogenic biomarkers and amniotic fluid at sampling at term

|  | **Spontaneous labour onset**  40.0 gw  **(n = 136)** | **Labour induction**  40.0 gw  **(n = 64)** | ***p* value** |
| --- | --- | --- | --- |
| PlGF (pg/ml) | 225 (143-401) | 203.5 (141-361) | 0.33 |
| sFlt-1 (pg/ml) | 3230 (2392-4638) | 3224 (2541-4633) | 0.96 |
| sFlt-1/PlGF ratio | 14 (7-29) | 16 (8-29) | 0.49 |
| Single deepest amniotic fluid pocket, cm | 4.8 (4.2-5.6) | 5.0 (4.0-6.2) | 0.48 |

Continuous variables are presented as median (interquartile ranges).

Abbreviations: gw, gestational weeks; PlGF, placental growth factor; sFlt-1, soluble fms-like tyrosine kinase-1.
