# Supplementary material for: Angiogenic Serum Biomarker Levels Are Related to Onset of Labour in Low‐Risk Term and Post‐Term Pregnancies: A Prospective Observational Cohort Study
Source: BJOG. 2026 Mar 27;133(9):1777–84. doi: 10.1111/1471-0528.70231 (PMC13419333; doi:10.1111/1471-0528.70231)
Supplement: Supplementary file 3 — Table S3: Pearson correlation analysis between individual time intervals and angiogenic biomarkers for spontaneous labour onset group and labour induction group. [file BJO-133-1777-s003.docx]

Table S3: Pearson correlation analysis between individual time intervals and angiogenic biomarkers for spontaneous labour onset group and labour induction group

| Time interval | n | PlGF | sFlt-1 | sFlt-1/PlGF ratio |
| --- | --- | --- | --- | --- |
| TTDs | 136 | r = 0.05  n.s. | **r = -0.18**  ***p* = 0.03** | **r = -0.19**  ***p* = 0.03** |
| TLOs | 126 | r = 0.05  n.s. | r = -0.17  n.s. | **r = -0.18**  ***p* = 0.046** |
| TLDs | 126 | r = -0.11  n.s. | r = -0.04  n.s. | r = -0.04  n.s. |
|  |  |  |  |  |
| TLIDi | 48 | **r = 0.34**  ***p* = 0.02** | r = -0.16  n.s. | r = -0.16  n.s. |
| TLOi | 46 | r = 0.18  n.s. | r = -0.16  n.s. | r = -0.11  n.s. |

Data are presented with correlation coefficient.

Abbreviations: r, Pearson correlation coefficient; *p*, *p* value; PlGF, placental growth factor; sFlt-1, soluble fms-like tyrosine kinase-1.

TTDs, total time to delivery (spontaneous labour onset group); TLOs, time interval to labour onset (spontaneous labour onset group); TLDs, time interval labour onset to delivery (spontaneous labour onset group);

TLIDi, time interval labour induction to delivery (labour induction group); TLOi, time interval labour induction to labour onset (labor induction group).
