# Supplementary material for: Angiogenic Serum Biomarker Levels Are Related to Onset of Labour in Low‐Risk Term and Post‐Term Pregnancies: A Prospective Observational Cohort Study
Source: BJOG. 2026 Mar 27;133(9):1777–84. doi: 10.1111/1471-0528.70231 (PMC13419333; doi:10.1111/1471-0528.70231)
Supplement: Supplementary file 4 — Table S4: Contingency table for cut‐off: sFlt1‐PlGF ratio 55. [file BJO-133-1777-s004.docx]

Table S4: contingency table for cut-off: sFlt1-PlGF ratio 55

| **Cut-off: sFlt1-PlGF ratio 55** | 2 days | 4 days | 7 days |
| --- | --- | --- | --- |
| Sensitivity (%) | **21**.**7** (9.7-41.9) | **14**.**3** (7.1-26.7) | **9**.**7** (5.2-17.4) |
| Specificity (%) | **94**.**7** (88.9-97.5) | **95**.**4** (88.8-98.2) | **95**.**4** (84.5-99.2) |
| Positive predictive value (%) | **45**.**5** (21.3-72.0) | **63**.**6** (35.4-84.8) | **81**.**8** (52.3-96.8) |
| Negative predictive value (%) | **85**.**6** (78.4-90.7) | **66**.**4** (57.7-74.1) | **32**.**8** (25.2-41.4) |
| Likelihood ratio | 4.1 | 3.1 | 2.1 |

Data are presented as percentages (95% confidence interval)

Abbreviations: PlGF, placental growth factor; sFlt-1, soluble fms-like tyrosine kinase-1.
